# International travel is not a significant risk of exposure for patients at a Midwestern United States travel clinic

**DOI:** 10.1186/s40794-020-00126-y

**Published:** 2020-12-10

**Authors:** Pooja Patel, Hans R. House

**Affiliations:** 1 Carver College of Medicine, University of Iowa, Iowa City, IA USA; 2 Department of Emergency Medicine, University of Iowa, 200 Hawkins Drive, RCP 1008, Iowa City, IA 52242 USA

**Keywords:** Coronavirus, International travel, Iowa, Transmission

## Abstract

The Novel Coronavirus (SARS-CoV-2) was introduced into the United States via travel from Asia and Europe, although the extent of the spread of the disease was limited in the early days of the pandemic. Consequently, international travel may have played a role in the transmission of the disease into Iowa. This study seeks to determine how preferences for international travel changed as novel Coronavirus Disease (COVID-19) spread throughout the world and if any of these returning travelers developed COVID-19 as a result of their trips. This is a retrospective chart review of patients presenting to a travel clinic in Bettendorf, Iowa for pre-travel advice and vaccinations. From October 2019 to March 2020, four hundred twelve (*n* = 412) patients presented to the clinic. Intended travel to the Western Pacific region (China, Japan, Korea, etc.) decreased dramatically during the study period. All 412 patients were followed in the electronic medical record for the period after their planned travel and only three (3) presented for COVID-19 testing. Two (2) tested positive, and both of these infections were linked to workplace exposures and not due to travel. News of the growing pandemic and travel warnings likely altered patients’ travel plans and decreased travel to the most affected regions of the world in the early months of the COVID-19 pandemic. Based on our study, travel was not a significant source of COVID-19 exposure for patients seen at this clinic.

## Introduction

In the early days of the COVID-19 pandemic, the Novel Coronavirus (SARS-CoV-2) rapidly spread worldwide, carried by international travelers via air transportation. SARS-CoV-2 was introduced into the United States due to travel from Asia and Europe, and it spread in the community for the first time in California and Washington [1]. Similarly, international travel may have played a role in the introduction of COVID-19 disease into Iowa, as the index cases were patients returning from a cruise in Egypt [2]. A travel medicine clinic in Bettendorf, Iowa offers pre-travel advice and immunizations to patients planning travel overseas. If imported disease was a major source of COVID-19 cases at the beginning of the pandemic in Iowa, then patients seen in the months prior to March 11, 2020 (the declaration of the pandemic by the World Health Organization (WHO)) would be at higher risk for illness. Additionally, the distribution of planned trips overseas for patients seen at the clinic as the pandemic unfolded may provide an insight into how attitudes towards travel changed as the disease spread worldwide. We expect that patients would alter or cancel their plans for international travel as reports of COVID-19 outbreaks occurred around the world. Therefore, our hypothesis is that patients planned less travel to the Western Pacific region (China, Korea, Japan, etc) as news of the novel coronavirus became more common between January and March 2020.

## Methods

This is a retrospective chart review of all patients presenting to a travel medicine clinic in Iowa from October 2019 to March 2020. The clinic closed for business on March 25th, 2020, due to low volumes of patient appointments and the risk of illness from local community transmission of COVID-19. The intended travel destinations for all patients were recorded and classified into each of the six World Health Organization (WHO) regions (Africa, Americas, Europe, Eastern Mediterranean, Western Pacific, and South-East Asia). Follow-up was conducted on each of these patients at least 60 days following their clinic visit. The electronic health record (EHR) (EPIC, Verona, WI) for the University of Iowa Hospitals and Clinics (UIHC) was accessed and each patient was searched for a report of a COVID-19 test. UIHC is the local referral hospital for the area and handled the majority of COVID-19 testing in the early months of the epidemic, so any new cases of COVID-19 disease during January to March 2020 should have been included in this EHR. Furthermore, most hospitals in the area use EPIC, and the EHR at UIHC is capable of viewing medical data from non-UIHC institutions using EPIC. The project was reviewed by the institutional review board (IRB) of the University of Iowa, and it was determined to not be human subjects research. However, as a condition of this approval, the IRB did not permit contacting individual patients from the chart review for a more detailed follow-up.

## Results

**Fig. 1:**
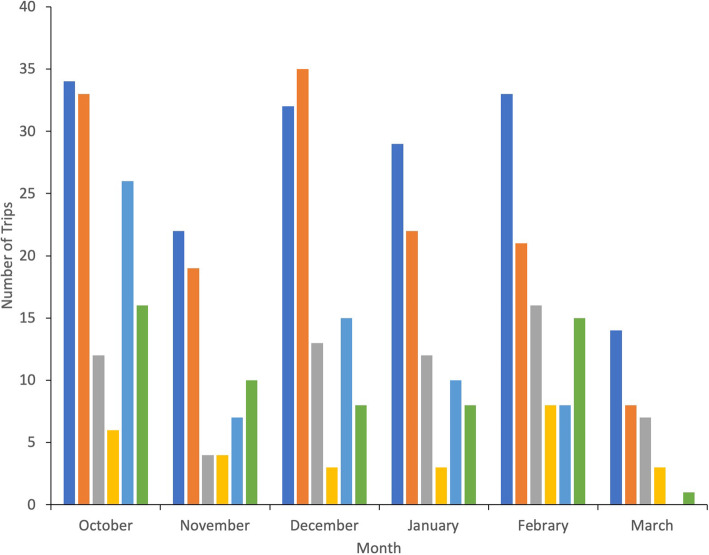
Number of trips planned to each WHO region by month, 2019–2020. The columns show the number of trips planned to each region by month of travel clinic visit. The regions are  Africa,  Americas,  Europe,  Eastern Mediterranean,  Western Pacific, and South-East Asia

From October 1st, 2019 to March 25th, 2020, four hundred and twelve (*n* = 412) patients ranging in age from one to eighty-two (mean = 42 years) presented for pre-trip guidance, vaccines, and prophylactic medications. Patients had planned travel throughout all six regions of the with an average of 22% of patients planning travel to multiple WHO regions in a given month (Fig. 1). In October 2019, 26 patients intended to travel to the Western Pacific region (i.e. China, Japan, Korea, etc.) while in March 2020, 0 patients planned on visiting this region. Furthermore, before January 2020, 11 patients planned travel to China, while in January 2020, only 3 patients planned to travel to China. In February and March 2020, no patients planned travel to China. Of the 412 patients, 118 appeared in the EHR for visits to UIHC and its many satellite clinics at some point after their travel clinic appointment. Three (3) patients were tested for COVID-19 and two (2) tested positive. Both positive tests were determined to be due to a workplace exposure through meat packing plants, and they were not related to a history of international travel.

## Discussion

Based on the chart review conducted in this study, imported cases of COVID-19 from international travel were not a significant component of the epidemic in Iowa. Only 2 of the 412 patients seen at the travel clinic from October 2019 to its closing in March 2020 tested positive for the disease, and neither of these cases were related to travel. Although the index cases in Iowa were returning travelers, widespread community disease became the predominant means of spread following this initial introduction. There is evidence that workers at meatpacking plants were a major amplifier of the epidemic in Iowa [3], and the two patients testing positive in this chart review had both been exposed through their work in food plants. To date, a scientific study of the outbreaks in Iowa’s meat packing faculities has not been published. The Centers for Disease Control conducted an epidemioligical review of COVID-19 disease in meatpacking plants, but the Iowa Department of Public Health declined to participate, so Iowa was not included in the study [4].

This study also examined patients’ planned travel preferences over time and provides a unique glimpse into the evolving anxiety and limitations on travel as the pandemic developed. Intended travel to the Western Pacific region (i.e. China, Japan, Korea, etc.) dropped precipitously throughout the study period. This change in planned travel preferences is likely attributable to the news of the emerging COVID-19 epidemic and the imposition of a level 4 travel warning (“Do Not Travel”) to China on February 2, 2020 by the U.S. Department of State. During this same time, travel to all other WHO regions remained fairly constant, followed by an abrupt reduction in March 2020. Notably, on March 11, 2020, COVID-19 was officially declared a pandemic by the World Health Organization (WHO), and most travel bans were implemented during this month.

This study is limited by utilizing one EHR for the follow-up on patient outcomes, as it is possible that the patients visited one of the few health centers in the region not using EPIC. Most patients in Iowa with COVID-19 in the early weeks of the epidemic would have been referred to UIHC so it unlikely a case in Iowa was missed. However, the travel clinic in the study draws patients from the neighboring state of Illinois, and it is possible that patients in that state would have visited a different hospital system or referral center. The dataset used for this study does not include state of residence. Additionally, the reason for travel (tourism, visiting friends and relatives, business, etc.) is not recorded in this dataset. It would have been interesting to see if the reasons for travel changed to any region as the pandemic developed, but that information was not available to the researchers.

It is apparent from the data that plans for travel decreased in the first few months of 2020. We are unable to compare this pattern to the same time in 2019, as the clinic opened for the first time in July of 2019. It is also possible that patients cancelled their previous planned travel during this time period, as more international travel warnings and restrictions were put into place. Data from the Transportation Security Administration (TSA) shows that airport traffic (measured in persons screened at security checkpoints in USA airports) remained stable until the second week of March 2020, when it rapidly decreased [5]. If the patients previously seen in clinic cancelled their travel plans, then they would not site international travel as a cause for their exposure to COVID-19. Additionally, this would make them less likely to seek testing for COVID-19, which may further depress the low number of positive cases seen in the follow-up for this patient data set.

## Conclusions

In closing, it is apparent that until March 2020, almost all travel behavior remained constant despite the first US case arising in mid-January. Patients at this clinic in Iowa altered their travel plans, but only late in the development of the pandemic. Fortunately, beyond the index cases, according to this study, travel did not seem to play an important role in COVID-19 disease in Iowa.

## Data Availability

The data used in this study were obtained from a travel clinic in Bettendorf, Iowa. It is available from the corresponding author on reasonable request.

## Abbreviations

SARS-CoV-2: Novel Coronavirus
COVID-19: Coronavirus Disease
WHO: World Health Organization
EHR: Electronic Health Record
UIHC: University of Iowa Hospitals and Clinics
IRB: Institutional Review Board
CDC: Centers for Disease Control
